# The Novel ncRNA OsiR Positively Regulates Expression of *katE2* and is Required for Oxidative Stress Tolerance in *Deinococcus radiodurans*

**DOI:** 10.3390/ijms21093200

**Published:** 2020-04-30

**Authors:** Lihua Gao, Xiaonan Chen, Ye Tian, Yongliang Yan, Yuhua Zhan, Zhengfu Zhou, Wei Zhang, Min Lin, Ming Chen

**Affiliations:** Biotechnology Research Institute, Chinese Academy of Agricultural Sciences, Beijing 100081, China; gaolihua89@hotmail.com (L.G.); 15933529573@163.com (X.C.); yezity114@163.com (Y.T.); yanyongliang@caas.cn (Y.Y.); zhanyuhua@caas.cn (Y.Z.); zhouzhengfu@caas.cn (Z.Z.); zhangwei01@caas.cn (W.Z.); linmin57@vip.163.com (M.L.)

**Keywords:** *Deinococcus radiodurans*, small noncoding RNA, OsiR, oxidative stress tolerance, *katE2* mRNA

## Abstract

*Deinococcus radiodurans* is a polyextremophilic bacterium well known for its extreme resistance to irradiation, oxidative stress, and other damaging conditions. Many small noncoding RNAs (ncRNAs) in *D. radiodurans* have been identified by deep sequencing analysis and computational predictions. However, the precise roles of ncRNAs and their target genes in the oxidative stress response have not been investigated. Here, we report the identification and characterization of a novel ncRNA named OsiR (for oxidative stress-induced ncRNA). Oxidative stress tolerance analysis showed that deleting *osiR* significantly decreased viability, total antioxidant capacity, and catalase activity in *D. radiodurans* under oxidative stress conditions. Comparative phenotypic and qRT-PCR analyses of an *osiR* mutant identify a role of OsiR in regulating the expression of the catalase gene *katE2*. Microscale thermophoresis and genetic complementation showed that a 21-nt sequence in the stem–loop structure of OsiR (204–244 nt) directly base pairs with its counterpart in the coding region of *katE2* mRNA (843–866 nt) via a 19 nt region. In addition, deletion of *katE2* caused a significant reduction of catalase activity and oxidative stress tolerance similar to that observed in an *osiR* mutant. Our results show that OsiR positively regulates oxidative stress tolerance in *D. radiodurans* by increasing the mRNA stability and translation efficiency of *katE2*. This work provides a new regulatory pathway mediated by ncRNA for the oxidative stress response that most likely contributes to the extreme tolerances of *D. radiodurans.*

## 1. Introduction

The polyextremophilic bacterium *Deinococcus radiodurans* can survive under high-intensity ionizing radiation, ultraviolet radiation, desiccation, and oxidative stresses [1,2,3,4]. Recent studies have shown that the strong resistance of *D. radiodurans* to various damaging conditions can be explained by its highly efficient antioxidative cell protection [4]. Indeed, strong resistance to oxidative stress plays an important role in the environmental adaptability [4,5]. Oxidative stress results from the formation of reactive oxygen species (ROS), of which the three main types are hydroxyl radicals, superoxide radicals, and hydrogen peroxide [6]. The ROS produced by ionizing radiation, UV radiation, desiccation, and oxidative stresses can damage proteins, lipids, nucleic acids, and carbohydrates and induce potentially lethal double-strand DNA breaks (DSBs) in the bacterial genome [7,8,9]. Therefore, it is not surprising that this bacterium has, over a long period of evolution, developed an effective antioxidant system to cope with oxidative stress. *D. radiodurans* displays pronounced resistance to all ROS-generating agents [10,11], and its ROS-scavenging enzymes include two major antioxidant enzymes: superoxide dismutase, which eliminates superoxide radicals from the cells, and catalase, which degrades H_2_O_2_. It has been reported that the ability of protein extracts of *D. radiodurans* to scavenge H_2_O_2_ was 30 times higher than that of *E. coli* extracts [12]; moreover, the catalase activity of *D. radiodurans* was 127 times and 32 times higher than that of *E. coli* during the exponential and stationary phases, respectively [13]. The *D. radiodurans* genome encodes two KatE-type catalases, DR1998 (KatE1), and DRA0259 (KatE2), and one eukaryotic-type catalase, DRA0146 [4]. Among these catalases, the typical monofunctional heme catalase DR1998 is the major catalase in *D. radiodurans*, and its expression is positively regulated by the transcriptional regulators DrRRA [14], OxyR [15], and PprM [16]. KatE2 also has extensive catalase activity, whereas DRA0146 does not exhibit catalase activity [17]. However, the biological functions and regulation of *katE2* have not yet been investigated in detail.

Bacterial regulatory noncoding RNA (ncRNAs), also referred to as small regulatory RNAs (sRNAs), are the core component of the bacterial stress response network and are multifunctional regulators that allow bacteria to adapt to a complex environment [18]. Many ncRNAs are involved in the regulation of diverse physiological processes, such as the cell division process [19], stress responses [20], quorum sensing [21], and bacterial virulence [22,23]. At present, most ncRNAs that have been characterized are *trans*-encoded and play a positive or negative role in mRNA stability and translation efficiency [24,25,26]. As ncRNA-based regulation is hypothesized to be dynamic and efficient, it represents a useful mechanism for polyextremophilic organisms to adapt to various survival stresses.

At present, with the rapid development of advanced bioinformatics prediction and sequencing technology, more and more ncRNAs have been identified in almost all bacteria [27,28]. Up to now, most of the ncRNAs which have been characterized in physiological functions and regulatory mechanisms originated from intestinal bacteria, such as *E. coli* and *Salmonella* [24,29]. The oxidative stress-induced sRNA OxyS from *E.coli* was the first characterized ncRNA involved in oxidative stress response [30], and has a negative regulatory effect on the mRNAs of transcription factors such as FhlA, RpoS, and FlhDC, as well as some other proteins [31,32]. Recent studies revealed that an OxyS-induced molecular checkpoint relay leads to temporary cell cycle arrest to facilitate DNA damage repair, thereby increasing viability following stress [33]. The *Pseudomonas stutzeri* ncRNA NfiS positively regulates the oxidative stress response by targeting a catalase-encoding gene, *katB* [34]. However, the detailed function and regulation of ncRNAs in the *Deinococcus* genus are only beginning to be studied, even though the first ncRNA of *D. radiodurans* similar to Y RNA was discovered nearly 19 years ago [35,36]. The binding complexes of Y RNA and RSR, a Ro protein ortholog, contribute to UV irradiation resistance. At present, 41 potential ncRNAs have been identified in *D. radiodurans* by combining deep sequencing analysis and computational predictions [37], but their biological functions and precise molecular mechanisms have not been characterized in detail except for Dsr18 (DnrH), which positively influences heat tolerance by increasing the transcription of *hsp20* mRNA [38]. One ncRNA named Dsr39 exhibited at least a 2-fold decrease in band intensity after 15-kGy irradiation analyzed by Northern blot [37]. In this study, we found that Dsr39 was highly expressed under oxidative stress induced by hydrogen peroxide, and the disruption of this ncRNA rendered *D. radiodurans* more sensitive to oxidative stress. Therefore, this ncRNA was renamed OsiR (oxidative stress-induced ncRNA). Our results indicate that OsiR act as a crucial regulator to positively regulate the oxidative stress resistance of *D. radiodurans* by directly base pairing with *katE2* mRNA, a catalase gene. The significant roles of OsiR in the oxidative stress response highlight the importance of ncRNA-mediated regulation under stress conditions in the *Deinococcus* species.

## 2. Results

### 2.1. Expression of osiR Is Induced by Oxidative Stress

**Figure 1:**
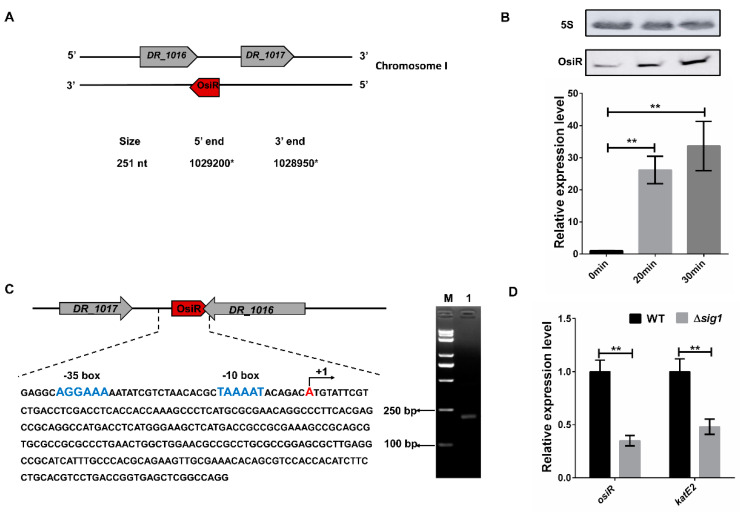
Expression and genomic context of OsiR. (**A**) the genomic location of *osiR* on chromosome I [37]; (**B**) transcript levels of *osiR* detected by Northern blot and qRT-PCR in *D. radiodurans*. 0 min, 20 min, 30 min indicates the time of H_2_O_2_ treatment; (**C**) physical map and nucleotide sequence of *osiR* in *D. radiodurans*. (blue) −10 box and −35 box in the promoter of OsiR predicted by BPROM; (+1) transcription start site mapped by 5’RACE; M: Trans2K PlusII Marker; Lane 1: product of the second nested PCR for 5’RACE; (**D**) effect of a *sig1* mutation on the expression of *osiR* and *katE2*. The qRT-PCR data shown are the averages of three biological replicates. Error bars represent standard deviations. The statistical significance of the difference was confirmed by Student’s *t*-tests (**, *p* < 0.01).

OsiR, which was initially identified in a previous report [37], is located in the intergenic region between *DR_1016* and *DR_1017* on chromosome I of *D. radiodurans*, with the 3’end overlapping with the 3’end of *DR_1016* by 79 bp (Figure 1A). *DR_1016* encodes a tRNA-modifying GTPase, and *DR_1017* encodes an rRNA methylase. qRT-PCR and Northern blot analyses revealed that expression of *osiR* was significantly upregulated under oxidative stress conditions (Figure 1B), suggesting that it may play a physiological role in the oxidative stress response.

**Figure 2:**
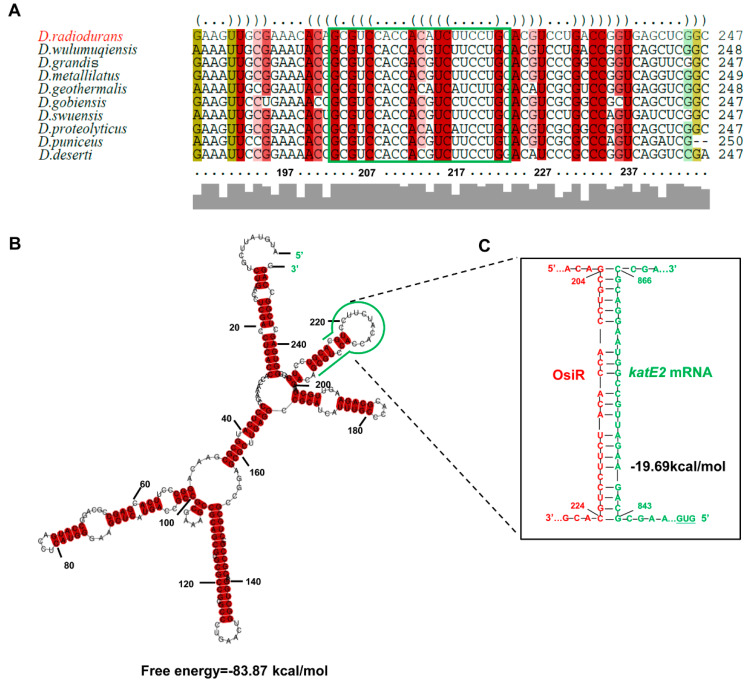
Sequence conservation and structural features of OsiR. (**A**) multisequence alignment of *osiR* homologous genes analyzed by LocARNA [39]. The colors indicate that the structural conservation, compatible base pairs are colored; the saturation decreases with the number of incompatible base pairs. Thus, it indicates the structural conservation of the base pair, for which red indicates 100% of the structure sequences is identical. (**B**) The secondary structure of OsiR predicted by RNAalifold [40]. The potential interaction region of OsiR with *katE2* mRNA is shown in green. The secondary structure stability of ncRNA is represented by the free energy values. (**C**) The interaction between OsiR and *katE2* mRNA analyzed by IntaRNA. OsiR is shown in red, and the *katE2* mRNA is shown in green.

The full-length *osiR* is 251 nt [37]; the transcription start site of *osiR* determined by 5’RACE is an adenine A at position 1,029,200 of chromosome 1 (Figure 1A, C). In the promoter region of *osiR,* a −10 box and a −35 box were predicted by BPROM, as shown in Figure 1C; these motifs are usually recognized and bound by the σ^70^ factor. The results of qRT-PCR assay showed that the transcript level of *osiR* was significantly decreased in the absence of *sig1*, suggesting that the expression of *osiR* may be controlled by Sig1, a σ^70^ factor in *D. radiodurans* (Figure 1D). Multi-sequence alignment of *osiR* and its homologs underscores the high degree of conservation in a region of 169–251 nt (Figure 2A), implying that this region likely plays the same role among members of *Deinococcus*. The predicted secondary structure of OsiR contains two stem–loop structures in this region (Figure 2B). These results indicated that OsiR is a *Deinococcus*-specific regulatory ncRNA induced by oxidative stress and may play a positive role in oxidative stress response.

### 2.2. OsiR Contributes to the Oxidative Stress Tolerance in D. radiodurans

**Figure 3:**
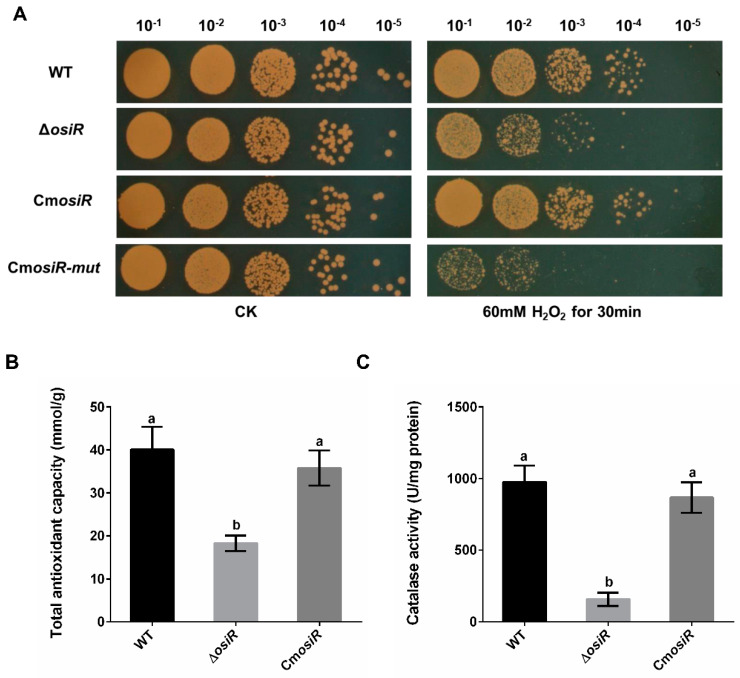
Deletion of *osiR* reduces the oxidative stress tolerance in *D. radiodurans*. (**A**) survival phenotype plate assay under oxidative stress conditions (one of three independent replicates). Serial 10-fold dilutions of OD-standardized cultures were spotted onto TGY plates before and after exposure to 60 mM H_2_O_2_. WT, wild type; Δ*osiR*, osiR deletion mutant; Cm*osiR*, complementary strain carrying wild-type *osiR*; CmosiR-mut, complementary strain carrying mutant *osiR*; CK, untreated culture control. Experiments were performed in triplicate. (**B**) and (**C**) effect of *osiR* mutation on the total antioxidant capacity and catalase activity of *D. radiodurans*. The values shown were derived from three independent experiments and error bars represent standard deviations. The statistical difference among the three samples (WT, Δ*osiR*, Cm*osiR*) was determined by Tukey multiple comparisons (*p* < 0.05); the same letter indicates that the difference is not significant and different letters indicate significant differences.

To further characterize the physiological role of OsiR in *D. radiodurans* under oxidative stress, the wild-type strain (WT), the *osiR*-deletion mutant strain (Δ*osiR*), and complementation strain (Cm*osiR*) were exposed to 60 mM H_2_O_2_ for 30 min. Under normal growth conditions (CK), there was no significant difference in the survival status of these strains; however, under oxidative stress conditions (60 mM H_2_O_2_ for 30 min), the survival ability of Δ*osiR* decreased significantly compared to WT, while Cm*osiR* had a similar survival ability to WT (Figure 3A). To further quantify the oxidative stress resistance of OsiR in *D. radiodurans*, the total antioxidant capacity (Figure 3B) and catalase activity of these strains (Figure 3C) were determined under oxidative stress; the results indicated that both total antioxidant capacity and catalase activity were significantly decreased in the absence of *osiR*. These results suggest that this ncRNA, as a positive regulator, plays an important role in the oxidative stress resistance in *D. radiodurans*, thus prompting us to search for target genes related to the oxidative stress response.

### 2.3. katE2 Is a Target of OsiR in Response to Oxidative Stress

**Figure 4:**
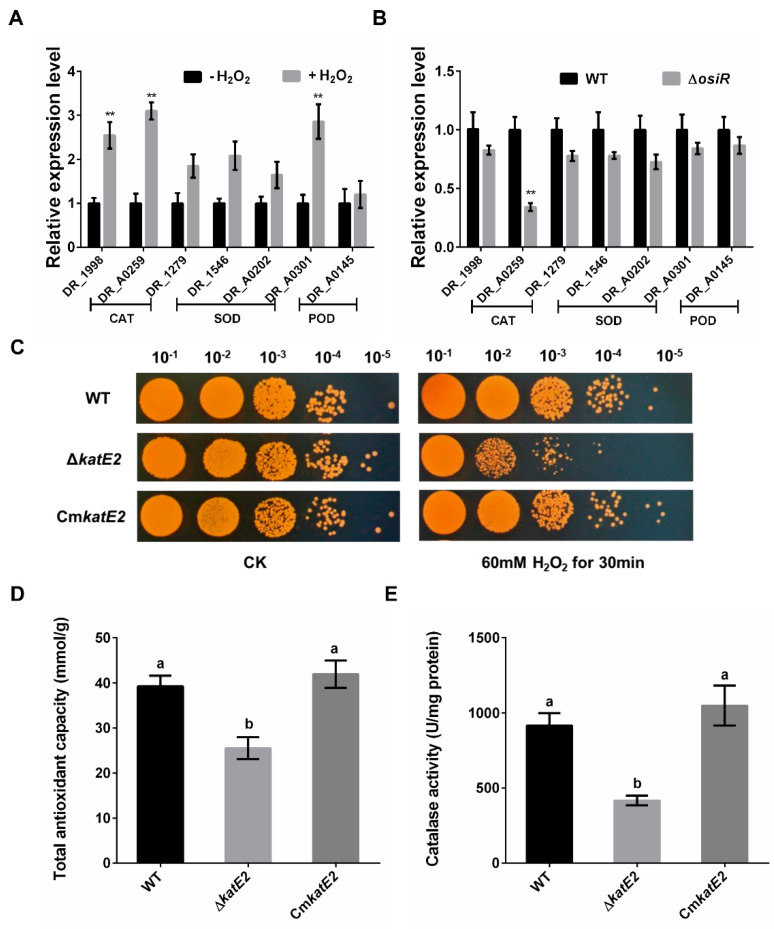
Regulation of OsiR on genes related to oxidative stress resistance and functional analysis of *katE2* under oxidative stress. (**A**) expression of seven selected oxidative stress–response genes of *D. radiodurans* grown in TGY with or without H_2_O_2_; (**B**) transcript levels of these genes of WT and Δ*osiR* strains grown in TGY with H_2_O_2_. All experiments were performed three times, and the results are represented as the mean ± standard deviation. Statistical analyses were performed using a Student’s *t*-test. The statistical significance is indicated as follows: **, *p* < 0.01; (**C**) survival phenotype plate assay of WT, Δ*katE2* and Cm*katE2* under oxidative stress conditions; (**D**) and (**E**) effect of *katE2* deletion on the total antioxidant capacity and catalase activity of *D. radiodurans*. The values shown were derived from three independent experiments, and error bars represent standard deviations. The statistical difference among the three samples (WT, Δ*osiR*, Cm*osiR*) was determined by Tukey multiple comparisons (*p* < 0.05); the same letter indicates that the difference is not significant and different letters indicate significant differences.

To identify potential targets of OsiR, qRT-PCR was used to determine the transcript levels of oxidative-related genes in *D. radiodurans* under oxidative stress conditions. The data showed that 3 genes, including *katE2*, exhibited a significant increase in expression after H_2_O_2_ shock (Figure 4A); however, only the *katE2* transcript level was significantly downregulated in Δ*osiR* compared to that in WT (Figure 4B), suggesting that the expression of *katE2* is OsiR dependent via an unknown mechanism. In addition, in silico (IntaRNA) analysis revealed a putative interaction between a typical stem–loop structure of OsiR (204–224 nt) and the CDS of the *katE2* mRNA (843–866 nt), with an interaction energy of −19.69 kcal/mol (Figure 2C). Thus, it was speculated that *katE2* is a potential target of OsiR under oxidative stress.

*katE2* encodes a KatE-type catalase that can protect cell components from oxidative damage under various stress conditions [11,16]. To investigate the biological function of *katE2*, a *katE2*-deletion mutant strain (Δ*katE2*) and complementary strain (Cm*katE2*) were constructed, and oxidative stress tolerance was determined. The phenotypic results showed that Δ*katE2* exhibited higher sensitivity to oxidative stress than WT (Figure 4C). Consistent with the phenotypic results, the disruption of *katE2* resulted in a significant decrease in the total antioxidant capacity and catalase activity in *D. radiodurans* (Figure 4D,E). In addition, expression of *katE2* is also significantly decreased in Δ*sig1* deletion mutant (Figure 1D). Taken together, these data confirmed that *katE2* is a potential target of OsiR and that the loss of *katE2* significantly decreased the oxidative stress tolerance in *D. radiodurans*.

### 2.4. OsiR Positively Regulates the Expression of katE2

**Figure 5:**
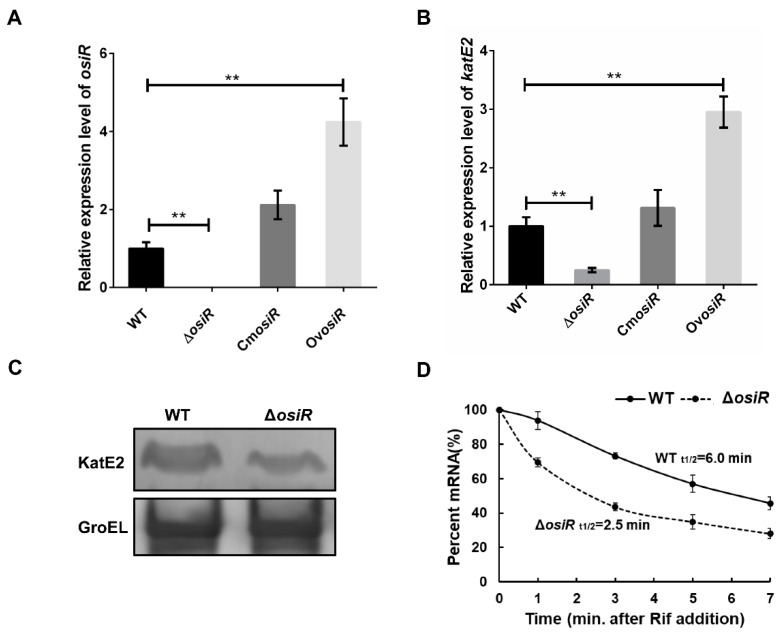
Deletion of *osiR* leads to a decrease in *katE2* expression. (**A**) and (**B**) qRT-PCR analysis of *osiR* and *katE2* in WT, Δ*osiR*, Cm*osiR*and Ov*osiR*. All experiments were performed three times, and the results are represented as the mean ± standard deviation. Statistical analyses were performed using a Student’s *t*-test. Statistical significance is indicated as follows: **, *p* value < 0.01. (**C**) Western blot analysis of the KatE2 protein in WT and Δ*osiR*. GroEl protein served as a loading control; (**D**) determination of the *katE2* mRNA half-life in WT and Δ*osiR* under oxidative stress conditions. The data shown are the averages of three biological replicates. Error bars represent standard deviations.

Positive regulation by ncRNA often occurs through the stabilization of transcripts, which leads to increased translation [41]. To further determine whether the presence of OsiR leads to an increased abundance of *katE2* transcripts, the relative expression levels of *osiR* and *katE2* in WT, Δ*osiR*, Cm*osiR* and Ov*osiR* under oxidative stress (60 mM H_2_O_2_ for 30 min) were examined by qRT-PCR. The data indicated that the deletion of *osiR* resulted in a significant reduction in *katE2* transcript level, while the overexpression of *osiR* significantly increased the *katE2* transcript level (Figure 5A, B). Western blot analysis showed a significant reduction in the KatE2 protein level in Δ*osiR* (Figure 5C). In addition, mRNA stability analysis indicated that the half-life of *katE2* mRNA in WT and Δ*osiR* were 6.0 min and 2.5 min, respectively (Figure 5D). These results led to a hypothesis that, in the absence of OsiR, the *katE2* transcript is rapidly degraded, leading to a decreased KatE2 protein production and oxidative stress tolerance in *D. radiodurans*.

### 2.5. OsiR Directly Base Pairs With katE2 mRNA to Enhance Oxidative Stress Tolerance in D. radiodurans

**Figure 6:**
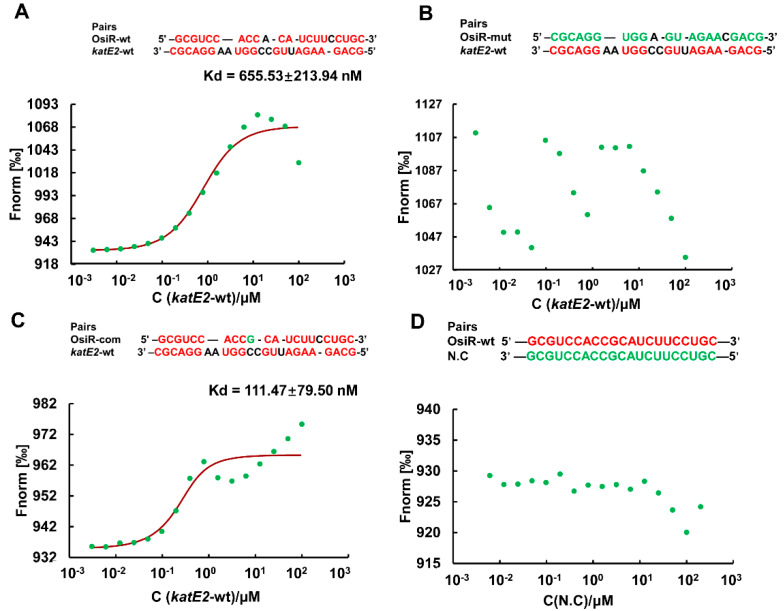
The binding affinity of OsiR to the *katE2* mRNA by MST analysis in vitro. Pairing nucleotides are shown in red. Point mutations introduced into synthesized oligonucleotide derivatives are shown in green. The K_d_ value reflects the binding affinity between two molecular fragments. wt: wild type; mut: mismatch mutation; com: compensatory mutation; N.C: negative control. (**A**) OsiR-wt/*katE2*-wt; (**B**) OsiR-mut/*katE2*-wt; (**C**) OsiR-com/ *katE2*-wt; (**D**) OsiR-wt/N.C

Taking the above results together, it was concluded that *katE2* is a target of OsiR during the regulation of oxidative stress tolerance in *D. radiodurans.* ncRNA-mediated regulation of gene expression usually occurs by direct base pairing, and the IntaRNA program predicted a ncRNA-mRNA base pairing interaction between OsiR and the *katE2* mRNA (Figure 2C). To verify this interaction, MST analysis, which allows for the sensitive measurement of molecular interactions in solution, was performed to detect the binding affinity between OsiR and the *katE2* mRNA. The MST results showed that OsiR-wt bound *katE2*-wt in a normal manner with a dissociation constant (K_d_) of 655.53 ± 213.94 nM, suggesting a strong interaction (Figure 6A). After replacing 19 bases in the base-pairing region of OsiR, there is no binding affinity between OsiR-mut and *katE2*-wt (Figure 6B). In addition, the OsiR-com containing compensatory mutations in the base-pairing region bound to *katE2*-wt in a high match level (K_d_ = 111.47 ± 79.50 nM) (Figure 6C), suggesting a stronger interaction. The negative control as shown in Figure 6D exhibited no binding curve between OisR-wt and N.C which contains the completely mismatched base complementary region with OsiR. Together, these data strongly suggest that OsiR is capable of interacting with the *katE2* mRNA via direct base pairing and the 19-nt of OsiR is very important for the binding of OsiR and *katE2* mRNA.

To further determine the effect of this base-pairing region of OsiR interacting with *katE2* mRNA as confirmed by MST on the physiological function of OsiR, the oxidative stress tolerance of the Δ*osiR* complemented with mutated *osiR* (Cm*osiR*-*mut*) was assessed. As shown in Figure 3A, the oxidative stress tolerance of Δ*osiR* was almost fully complemented by WT *osiR* but hardly complemented by the mutated *osiR*. These results strongly indicate that the 19-nt base-pair located on the stem–loop structure of OsiR, complementarity with *katE2* mRNA, is functionally required for OsiR to positively regulate the oxidative stress tolerance in *D. radiodurans*.

## 3. Discussion

*D. radiodurans* is unparalleled among all known organisms with regard to extreme resistance to oxidative stress-generating agents which affects all cellular macromolecules [7,10]. More than a hundred genes have been identified to contribute to oxidative stress tolerance in *D. radiodurans*; however, to date, the regulatory mechanism underlying the extreme oxidative stress tolerance in *D. radiodurans* has not been fully elucidated [5,11]. ncRNA-mediated regulation plays an important role in stress adaptation and gene regulation in almost all bacteria [20,42,43]. Furthermore, post-transcriptional regulation by ncRNAs enables bacteria to respond to the signals in an extremely flexible and sensitive manner [44,45]. Over the last few years, an increasing number of bacterial ncRNAs have been identified by combining RNA-sequences and bioinformatics [27,28,36]; however, until now, there are very few studies unravelling the biological roles and/or identifying the target genes of ncRNAs in extremophilic bacterium *D. radiodurans*. To our knowledge, the research on the specific molecular regulatory mechanism of ncRNA in regulating oxidative stress resistance in *D. radiodurans* has not been reported. Our work shows that OsiR could be a novel *Deinococcus-*specific ncRNA that positively regulates oxidative stress tolerance via direct base-pairing with the *katE2* mRNA in *D. radiodurans*. As indicated by Northern blot, qRT-PCR, and physiological phenotype analyses, the *osiR* gene was highly expressed under oxidative stress condition from a Sig1-dependent promoter. Moreover, deletion of *osiR* led to a significant decrease in oxidative stress tolerance and catalase activity in *D. radiodurans*.

KatEs are a kind of common catalase that detoxifies H_2_O_2_ widely distributed in *Deinococcus* genus (Table S1). *D. radiodurans* genomes encoded two KatE-type catalases (KatE1 and KatE2) to cope with oxidative stress [17]. Here, our results clearly demonstrate that *katE2* mRNA is a target of OsiR under oxidative stress condition and that the expression of *katE2* is positively regulated by OsiR at the posttranscriptional level. MST in vitro further demonstrated that the effect of OsiR on *katE2* expression was achieved through a direct interaction mediated by base pairing. Furthermore, secondary structure analysis of the *katE2* mRNA predicted a hairpin structure in the coding region downstream of the start codon, and such a hairpin structure is generally believed to affect translation efficiency. Interaction between OsiR with *katE2* mRNA promotes the ribosome binding and progression (Figure S1). This is a novel regulatory mechanism different from that of *katE1*, the expression of which is controlled at the transcriptional level by protein regulators such as DrRRA [14], OxyR, and PprM [16], suggesting that the coordination of *katE1* and *katE2* expression may be an efficient strategy for ROS scavenging in *D. radiodurans.* In addition, deletion of *katE2* also significantly decreased oxidative stress resistance as well as catalase activities in *D. radiodurans*. qRT-PCR analysis indicated that the transcription of *katE2* was significantly induced by oxidative stress, again confirming the role of *katE2* in oxidative stress tolerance.

**Figure 7:**
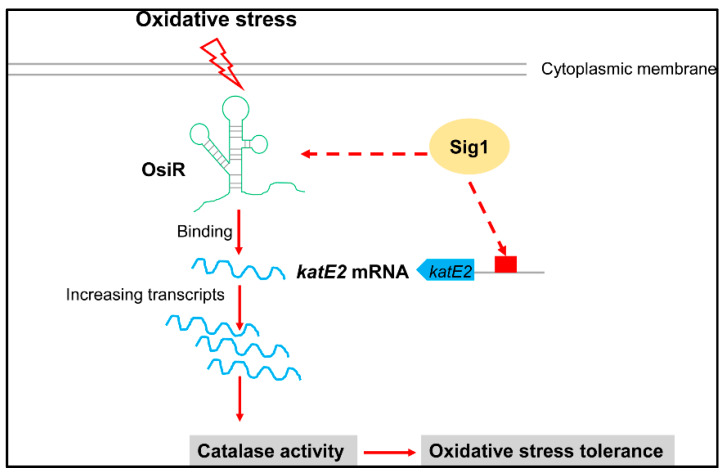
Model for the OsiR-mediated regulatory network in the oxidative stress response in *D. radiodurans*. OsiR acts as a riboregulatory to contribute to oxidative stress tolerance and catalase activity via direct base pairing with *katE2* mRNA or via indirect transcriptional activation of Sig1. For more details, see the results and discussion.

Taken together, our work indicates that OsiR is a novel ncRNA involved in the oxidative stress response in *D. radiodurans.* The working model as shown in Figure 7 illustrates the regulatory mechanism of OsiR with regard to how it regulates oxidative stress tolerance in *D. radiodurans*. At the transcriptional level of global regulation, the expression of *osiR* and *katE2* depends on Sig1; at the posttranscriptional level, OsiR promotes expression of *katE2* by increasing the stability of *katE2* mRNA, and ultimately enhances the catalase activity and oxidative stress tolerance of *D. radiodurans*. Whether OsiR directly regulates the expression of other oxidative-related genes under various physiological processes remains to be further explored. Future research should help us understand why this bacterium is extremely resistant to ionizing radiation and oxidative stress. Our work presented herein exemplifies the growing appreciation of the importance of ncRNAs in *Deinococcus* and further expands the list of ncRNAs known to play a role in oxidative stress response.

## 4. Materials and Methods

### 4.1. Bacterial Strains, Plasmids, and Growth Conditions

All bacterial strains and plasmids used in this study are described in Table 1. *D. radiodurans* was obtained from China General Microbiological Culture Collection Center (CGMCC 1.633, Beijing, China). *D. radiodurans* and derivatives were cultured in liquid TGY medium (1% tryptone, 0.1% glucose, and 0.5% yeast extract) at 30 °C or cultured on solid TGY medium with 1.5% agar. When required, kanamycin and chloromycetin were added to final concentrations of 20, 3.4 µg/mL, respectively. *E. coli* strains were cultured in LB medium.

### 4.2. Nucleotide Sequence

The primer sequences used in this study are shown in Table S2. The primers were synthesized by Shanghai Sangon Biotech, and the single-strand RNAs for MST were synthesized by GenePharma Company (Table S3).

### 4.3. Construction of the Deletion Mutants and Complementation Strains

The *osiR* and *katE2* deletion mutants were constructed by a double crossover recombination of a kanamycin (kan) resistance cassette into the genome as described previously [46,47]. PCR primers were designed according to the sequence of *osiR* in the genome. The primers OsiR-P1 and OsiR-P2 were used to amplify the upstream DNA fragment of *osiR* (432 bp). The kan resistance gene (*NptII*) was amplified from pKatAPH3 by the primers OsiR-P3 and OsiR-P4 (946 bp). The primers OsiR-P5 and OsiR-P6 were used to amplify the downstream DNA fragment of *osiR* (503 bp). The three amplified DNA fragments were used as templates for the overlap reaction and the resulting PCR fragment (1829 bp) was directly transformed into *D. radiodurans*. The single colony with kanamycin (20 µg/mL) resistance was selected. Finally, PCR and DNA sequencing were used to verify that the *osiR* gene was knocked out. The successfully constructed mutant was named Δ*osiR*.

DNA fragments containing the *osiR* gene and its promoter region were amplified by PCR. The PCR products were digested with *Hin*dIII/*Bam*HI and ligated to the plasmid pRADZ3 to generate the complementation plasmid pRADZ3-*osiR*. pRADZ3-*osiR* was directly transformed into Δ*osiR* and *D. radiodurans* to generate the complementary strain Cm*osiR* and the overexpressing strain Ov*osiR*, which were selected with 20 µg/mL kanamycin and 3.4 µg/mL chloramphenicol. The strains were confirmed by PCR and DNA sequencing. The *katE2*-deletion mutant and complementary strain were constructed in the same way.

### 4.4. Quantitative Real-Time PCR (qRT-PCR)

Total RNA was isolated with a PureLink RNA Mini Kit (Invitrogen, Thermo Fisher, Waltham, MA, United States) and was reverse-transcribed into cDNA with the PrimeScript^TM^ RT reagent kit. qRT-PCR was performed using ChamQ SYBR qPCR Master Mix (Vazyme Biotech, NanJing, China) with an AB7500 Fast Real-time PCR System (Applied Biosystems, Foster City, CA, USA). The 16S rRNA gene (*DR_r06*) was used as the endogenous reference control to normalize the expression of target genes in each cDNA sample. The relative expression levels of genes were calculated by the comparative threshold cycle (2^−ΔΔ*CT*^) method. All qRT-PCR experiments were performed with three biological replicates.

### 4.5. 5‘RACE 

The transcriptional start site of the *osiR* gene was determined using a 5’ rapid amplification of cDNA ends (5’RACE) kit (Roche, Mannheim, Germany) according to the manufacturer’s instructions. In short, total RNA was isolated, and the first-strand of cDNA was generated using a specific primer (GSP1) for the *osiR* gene. The original cDNA was purified and the 3’ end of the cDNA was tailed with dATP by the recombinant terminal transferase, and dA-tailed cDNA amplification was performed using the specific primer GSP2 and the anchor primer. Then, the second amplification round was performed using the specific primer GSP3. The PCR products were cloned into the pMD18-T vector (Takara Bio, Dalian, China) and sequenced to map the 5’ ends of the transcript. The primer sequences of GSP1, GSP2, and GSP3 are listed in Table S2.

### 4.6. Northern Blot Analysis

Northern blot was performed following the previous reports [38,48]. Total RNA was isolated from WT *D. radiodurans* grown under oxidative stress and normal conditions. The RNA samples (15 µg) were first denatured at 90 °C for 10 min and then separated on an 8% urea polyacrylamide gel and electroblotted onto a positively charged nylon membrane. Digoxigenin-labeled DNA probes (30 nt) were synthesized (Sangon Biotech, Shanghai, China) and used for hybridization overnight at 42 °C. The membrane was incubated in blocking buffer for 2–3 h at room temperature, placed in blocking buffer with an anti-DIG antibody for 30 min and washed in DIG washing buffer 3–4 times. Finally, the membrane was incubated in detection buffer for 5–15 min and in CSPD (chemiluminescence substrate) solution for 15–30 min in the dark. Band intensity analyses were performed using an Amersham imager 600 RGE (GE Healthcare, Pittsburgh, USA).

### 4.7. Western Blot Analysis

Western blot was performed as previously described [16]. To investigate the KatE2 protein level in WT and Δ*osiR* under oxidative stress conditions, bacterial cells (OD_600_ = 0.8, treated with 60 mM H_2_O_2_ for 30 min) were harvested by centrifugation at 4 °C for 7 min. The cells were washed twice and resuspended in 1 × PBS and disrupted ultrasonically on ice with an ultrasonic processor. The cell-free extracts were collected by centrifugation, and the protein concentration was determined using the Bradford colorimetric assay with bovine serum albumin (BSA) as the standard. A 20 µL aliquot of each sample was heated at 10 °C for 8 min, subjected to 12% SDS-PAGE and transferred onto a polyvinylidene fluoride (PVDF) membrane. The PVDF membrane was incubated with a rabbit anti-KatE2 polyclonal antibody (1:2500). *D. radiodurans* GroEL as a control was detected using *E. coli* GroEL antiserum (1:2500). Peroxidase-conjugated goat anti-rabbit IgG (1:5000) was used as the secondary antibody. Chemiluminescent signals on the PVDF membrane were visualized using an Amersham imager 600 RGE (GE Healthcare, Pittsburgh, USA).

### 4.8. Oxidative Stress Survival Assays

Oxidative stress survival assays were carried out according to previous methods [46,48]. Strains were grown overnight in TGY broth at 30 °C and were transferred into fresh TGY broth up to OD_600_ = 0.8; 60 mM H_2_O_2_ was added to the medium for 30 min. Next, 8 µL of serial 1 × PBS 10-fold dilutions of OD-standardized cultures were spotted onto TGY plates, which were incubated at 30 °C for 3 days before colony growth was observed. All the assays were performed in triplicate.

### 4.9. Half-Life Experiment

Measurement of the *katE2* mRNA half-life was performed as described previously [34]. To determine the half-life of *katE2* mRNA under oxidative stress, 60 mM H_2_O_2_ was added to TGY broth when cells were cultured to OD_600_ = 0.8. Rifampin (40 mg/mL) was added immediately after H_2_O_2_ treatment, and 2 mL samples were collected at different time points (0, 1, 2, 3, 4, and 5 min). Subsequently, 4 mL RNAlater (Sigma, Saint Louis, MO, USA) was added to protect RNA, and the samples were incubated at room temperature for 5 min and centrifuged for 2 min at 12,000 rpm. The pellets were quickly frozen in liquid nitrogen. Total RNA was isolated and cDNA was synthesized and used to estimate *katE2* mRNA levels by qRT-PCR. Data are presented as the percentage of *katE2* mRNA levels relative to the amount of these mRNAs at time point zero. The data are the mean of three biological repeats.

### 4.10. Determination of Total Antioxidant Capacity and Catalase Activity

The total antioxidant capacity and catalase activities of samples were determined using the total antioxidant capacity kit and catalase activity kit (Beyotime Institute of Biotechnology, Jiangsu, China) according to the instructions. The principle of the FRAP method for determination of total antioxidant capacity is that Ferric-tripyridyltriazine (Fe^3+^-TPTZ) can be reduced by antioxidants under acidic conditions to produce blue Fe^2^^+^-TPTZ, and then the total antioxidant capacity in the sample can be determined by measuring the blue Fe^2^^+^-TPTZ at 593 nm. All bacterial cells (OD_600_ = 0.8, treated with 60 mM H_2_O_2_ for 30 min) were harvested by centrifugation for 7 min at 12,000 rpm, washed and resuspended in 1 × PBS, and disrupted on ice with an ultrasonic processor. The clear supernatants were collected by centrifugation at 13,000× *g* at 4 °C for 15 min. Total protein concentrations were determined by the Bradford method using BSA as the standard. The next operation steps were completed according to the instructions of total antioxidant capacity test kit. The catalase activity assays were performed according to the kit protocol. Samples were prepared and added to a 96-well plate and incubated at 25 °C for 15–45 min, and the absorbance was determined at 520 nm. For total antioxidant capacity, a standard curve was generated using the FeSO_4_•7H_2_O provided in the kit. Next, the 180 µL FRAP solution was added to a 96-well plate, and different samples, control, and FeSO_4_ standard solution were added; the plates were incubated at 37 °C for 5 min, and absorbance was determined at 593 nm. The total antioxidant capacity of the sample was calculated according to the standard curve. The experiments were performed three times independently.

### 4.11. Microscale Thermophoresis Measurements (MST)

MST experiments were performed according to previous reports [34,48]. A set of full-length ncRNAs containing WT or derivative OsiR for MST was transcribed in vitro by GenePharma using a MAXIscript kit (Thermo Fisher, MA, USA). Another set of 6-FAM-labeled 70-nt single-stranded RNAs (ssRNAs) containing WT *katE2* mRNA (N- *katE2* competitors) was synthesized by GenePharma (GenePharma, Jiangsu, China), as listed in Table S3. Four microliters of sample containing 250 nM labeled probe and increasing concentrations of a non-labeled competitor (from 10 nM to 340 µM) were loaded into standard treated silicon capillaries (Monolith NT.115 series capillaries; catalog no. MO-K002). Measurements were carried out using a Monolith NT.115 instrument (NanoTemper Technologies, Munich, Germany) at 25 °C in diethyl pyrocarbonate (DEPC)-treated water with 40% excitation power and medium MST-Power. The dissociation constant (K_d_) was calculated as described previously [49]. Data analyses were performed using Nanotemper Analysis software (NanoTemper Technologies, Munich, Germany).

## 5. Patents

201810666398.0; 201810666544.X.

## Figures and Tables

**Table 1:** Strains and plasmids used in this study.

Strains/Plasmids	Relevant Characteristics	Source
***Deinococcus radiodurans***	Wild type	Lab stock
*osiR*	*D. radiodurans osiR-*deletion mutant, Km^r^	This study
*katE2*	*D. radiodurans katE2-*deletion mutant, Km^r^	This study
Cm*osiR*	*osiR* containing the complementation plasmid pRADZ3-*osiR*, Km^r^, Cm^r^	This study
Cm*osiR-mut*	*osiR* containing the complementation plasmid pRADZ3-*osiR*-*mut*, Km^r^, Cm^r^	This study
Cm*katE2*	*katE2* containing the complementation plasmid pRADZ3-*katE2*, Km^r^, Cm^r^	This study
Ov*osiR*	*D. radiodurans* containing the plasmid pRADZ3-*osiR*, Cm^r^	This study
***Escherichia coli***		
Trans 10 Trans 10 *Z3-osiR* Trans 10 *Z3-katE2* Trans 10 *Z3-osiR-mut*	Host for cloning vectors As trans 10 with pZ3*-osiR* As trans 10 with pZ3*-katE2* As trans 10 with pZ3*-osiR-mut*	TransGen This study This study This study
**Plasmids** *pRADZ3*	Shuttle plasmid between *E. coli* and *D. radiodurans*, ampicillin in *E. coli* chloromycetin in *D. radiodurans*	Lab stock
p*osiR-wt*	pRADZ3-derived plasmid carrying the wildtype *osiR* gene, Cm^r^, Amp^r^	This study
p*katE2-wt*	pRADZ3-derived plasmid carrying the wildtype *katE2* gene, Cm^r^, Amp^r^	This study
p*osiR-mut*	pRADZ3-derived plasmid carrying the mutant *osiR* gene, Cm^r^, Amp^r^	This study

## Supplementary Materials

Supplementary materials can be found at https://www.mdpi.com/1422-0067/21/9/3200/s1. Supplementary Figure S1: Schematic of the interaction between OsiR and *katE2* mRNA. Supplementary Table S1: Distribution of homologous genes of *katE1* and *katE2* in *Deinococcus* species. Supplementary Table S2: Primers used in this study. Supplementary Table S3: Synthesized ssRNA oligonucleotide derivatives for MST.
