# Supplementary material for: The Novel ncRNA OsiR Positively Regulates Expression of katE2 and is Required for Oxidative Stress Tolerance in Deinococcus radiodurans
Source: Int J Mol Sci. 2020 Apr 30;21(9):3200. doi: 10.3390/ijms21093200 (PMC7247583; doi:10.3390/ijms21093200)
Supplement: Supplementary file 1 [file ijms-21-03200-s001.pdf]

International Journal of
Molecular Sciences

Supplementary Materials: The Novel ncRNA OsiR Positively Regulates Expression of *katE2* and Is Required for Oxidative Stress Tolerance in *Deinococcus radiodurans*

1. Supplementary Tables

Table S1. Distribution of homologous genes of *katE1* and *katE2* in *Deinococcus* species.

Species	*katE1* of homologous genes (number)	Identity (%)	*katE2* of homologous genes (number)	Identity (%)
D. radiodurans	1		1	
D. wulumuqiensis	1	96.83	1	88.39
D. reticulitermitis	1	87.64	1	65.96
D. sp K2S05-167			1	61.52
D. sp.FeSDHB5-19			1	67.08
D. proteolyticus	1	85.91	1	58.70
D. radiophilus	1	84.62	1	59.08
D. ficus			1	60.40
D. irradiatisoli	1	78.22	1	58.36
D. sp H1			1	59.12
D. sp S14-83			1	58.79
D. sp leaf326	1	82.90		
D. sp S9	1	78.07		
D. geothermalis	1	78.07		
D. phoenicis	1	77.97		
D. sp YIM 77859	1	78.10		
D. metallitatus	1	76.65		
D. deserti	1	74.71		
D. aerius	1	74.95		
D. apachensis	1	72.62		
D. planocerae	1	72.61		
D. sp RL	1	77.71		
D. murrayi	1	77.71		
D. yavapaiensis	1	71.57		

Red indicates that the homologous genes of *katE1* and *katE2* coexist in the same bacteria.

Table S2. Primers used in this study.

Primer Name	Sequences (5'–3')	Purpose
osiR-P1	ACCGAGGACCTGATCGAGGCTGC	Δ*osiR* deletion mutant
osiR-P2	GAGTTTTTCTAATCATTTGCCCACGCAGAAGTTG	consturction
osiR-P3	GTGGGCAAATGATTAGAAAAACTCATCGAGCATCAAATG	
osiR-P4	GCTAAAATACAGACCATGGAGACCGAGGGCCCTTGACATT	
osiR-P5	CCTCGGTCTCCATGGTCTGTATTTTAGCGTGTTAG	
osiR-P6	GTTCCGTCGCGCCCGTCACTTCG	
katE2-P1	CGCCGCGCTCGCCATCAGTG	Δ*katE2* deletion mutant
katE2-P2	CAAGGGCCCTCGGTCTCCATGCAGAGCGCGGCCTTTTCGGGA	consturction
katE2-P3	TCCCGAAAAGGCCGCGCTCTGCATGGAGACCGAGGGCCCTTG	
katE2-P4	GCCACCTTGAGGACTAATGGTTTAGAAAAACTCATCGAGC	
katE2-P5	GCTCGATGAGTTTTTCTAAACCATTAGTCCTCAAGGTGGC	
katE2-P6	TGACAGACAGCGCCTCGGCG	
qosiR-F	GTCTGACCTCGACCTCACCAC	qRT-PCR
qosiR-R	GGCAAATGATGCGGCCTCAAGC	
qkatE2-F	TGGGCAAGATGGTCCTCG	
qkatE2-R	CAGCGGGTCGTTGGTGA	
qkatE1-F		
qkatE1-R		
qDR_1279-F		
qDR_1279-R	ACCAACATCCAGTCGCAG	
qDR_1546-F	CCTTCACCCTGGTCGTTG	
qDR_1546-R	CCGCACGATGGAAATTCACC	
qDR_A0202-	CAGAACATGCTGTGGTTGGC	
F	CTTTAGCCCCAGTCCCATCG	
qDR_A0202-	ACCGTAATCATCGGCAGGTC	
R	CTGAACCGCAGTCTGGTCAT	
qDR_A0301-	AAGCCTTTCTTCGCGTCGTA	
F	TCGACAAGGCCGAGTGTTTT	
qDR_A0301-	GAGGCCGATGTCTTCGTTGA	
R	GACGATATTCAGGCGACCGT	
qDR_A0145-	CCACTTTGAACGACTCGGGA	
F		
qDR_A0145-		
R		
q16S-F	ATTCCTGGTGTAGCGGTG	
q16S-R	CATCGTTTAGGGTGTGGAC	
5S-NP	Dig-5'GTATCATTGGCGCTGCTGTGTTTCACGACCCAGTT	Northern blot
OsiR-NP	Dig-5'TGTATTCGTCTGACCTCGACCTCACCACCAA	
GSP1	5'-GACGTGCAGGAAGATGTGGTGGACGCTGTGTTTC-3'	5'RACE
GSP2	5'-GTGTTTCGCAACTTCTGCGTGGGCAAATGATGC-3'	
GSP3	5'-CAAGCGCTCCGGCGCAGGCGGCGTTCCAGCCAG-3'	

International Journal of
Molecular Sciences

MDPI

Table A3. Synthesized ssRNA oligonucleotide derivatives for MST.

Name	Sequence (5'–3')	Relevant Characteristics
OsiR-wt	AUGUAUUCGUCUGACCUCGACCUCACCACCAAAGCCCUCAUGCGCGAAC AGGCCCUUCACGAGCCGCAGGCCAUGACCUCAUGGGAAGCUCAUGACCG CCGCGAAAGCCGCAGCGUGCGCCGCGCCCUGAACUGGCUGGAACGCCGC CUGCGCCGGAGCGCUUGAGGCCGCAUCAUUUGCCCACGCAGAAGUUGCG AAACACAGCGUCCACCACAUCUUCCUGCACGUCCUGACCGGUGAGCUCG GCCAGG	wild type (251 bp), interaction with *katE2*-wt
OsiR-mut	AUGUAUUCGUCUGACCUCGACCUCACCACCAAAGCCCUCAUGCGCGAAC AGGCCCUUCACGAGCCGCAGGCCAUGACCUCAUGGGAAGCUCAUGACCG CCGCGAAAGCCGCAGCGUGCGCCGCGCCCUGAACUGGCUGGAACGCCGC CUGCGCCGGAGCGCUUGAGGCCGCAUCAUUUGCCCACGCAGAAGUUGCG AAACACACGCAGGUGGAGUAGAACGACGACGUCCUGACCGGUGAGCUCG GCCAGG	mismatch mutation (251 bp), no binding affinity with *katE2*-wt
OsiR-com	AUGUAUUCGUCUGACCUCGACCUCACCACCAAAGCCCUCAUGCGCGAAC AGGCCCUUCACGAGCCGCAGGCCAUGACCUCAUGGGAAGCUCAUGACCG CCGCGAAAGCCGCAGCGUGCGCCGCGCCCUGAACUGGCUGGAACGCCGC CUGCGCCGGAGCGCUUGAGGCCGCAUCAUUUGCCCACGCAGAAGUUGCG AAACACAGCGUCCACCGCAUCUUCCUGCACGUCCUGACCGGUGAGCUCG GCCAGG	compensatory mutation (251 bp), stronger interaction with *katE2*-wt
katE2-wt	UGCUCGGCGUCAAGAGCCUGGUGUGGGACGAAGCGCAGAAGAUUGCCG GUAAGGACGCCGACUUCCACCG	wild type (70 bp), interaction with osiR-wt
N.	UGCUCGGCGUCAAGAGCCUGGUGUGGGACGAAGCGCGUCCACCACAUCU UCCUGCCTACGACUUCCACC	ompletely mismatched (70 bp), no binding with osiR-wt

wt: wild type; mut: mutation; com: supplementary mutation; N.C: negative control. The base pairing region of OsiR with *katE2* mRNA is shown in red. Point mutations introduced into synthesized oligonucleotide derivatives are shown in green.

International Journal of
Molecular Sciences

2. Supplementary Figure

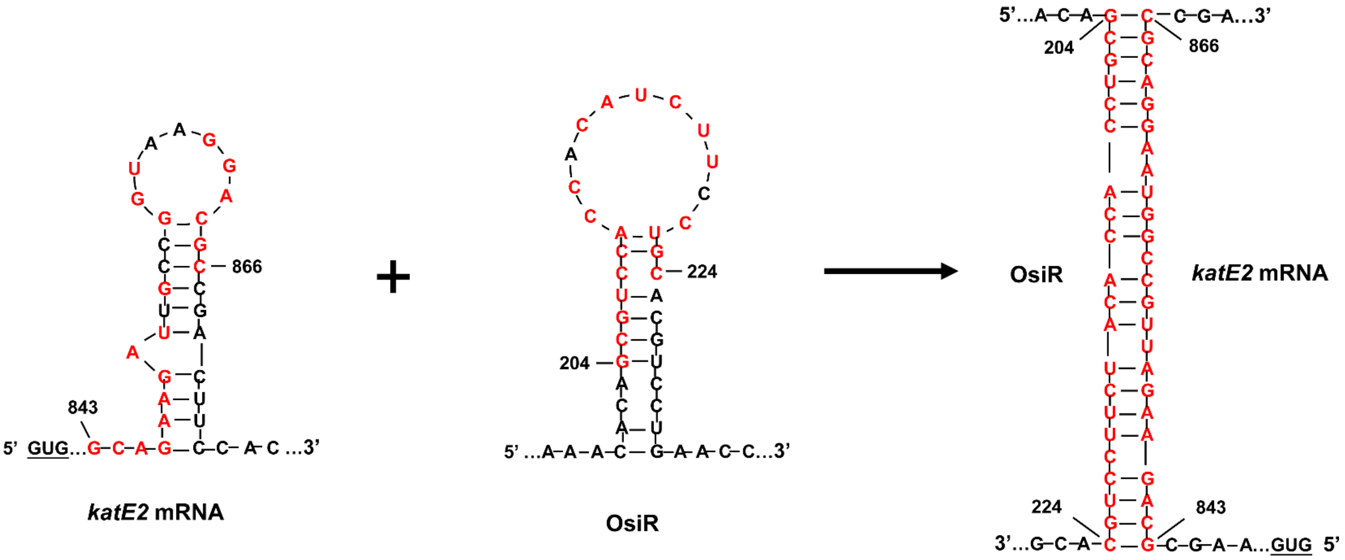

Figure S1. Schematic of the interaction between OsiR and *katE2* mRNA. The secondary structure of the *katE2* mRNA contains a putative hairpin structure (nucleotides 846–873), which includes the OsiR binding site, presumably forming an inhibitory structure that affects translation efficiency. The binding of OsiR and *katE2* mRNA promotes the ribosome binding and progression.
